# The transformative cognition of English as a Foreign Language student teachers’ personal practical knowledge

**DOI:** 10.3389/fpsyg.2023.1263552

**Published:** 2023-10-10

**Authors:** Quan Xu, Ting Wang

**Affiliations:** School of Foreign Languages, Central China Normal University, Wuhan, China

**Keywords:** practical knowledge, transformative cognition, blended learning community, filter mechanism, course expectation types, dynamic interaction

## Abstract

This study investigates the process by which English as a Foreign Language (EFL) student teachers transmute their Pedagogical Content Knowledge (PCK) into Personal Practical Knowledge (PPK) within a blended learning community. Data sources, including conversation transcripts, reflective journals, and field notes, were meticulously examined utilizing the commonplaces of temporality, sociality, and place. Several key findings were unveiled: (1) the volume and focal points of transformed PPK varied across participants; (2) the metamorphosis of PCK into PPK was found to be selectively partial, filtered by factors such as previous learning experiences, course expectations, and levels of engagement; and (3) the selection process was molded within the dynamic interplay of the internal components of the blended learning community and the external socio-cultural conditions. The study concludes that the cognition mechanism of EFL student teachers’ PPK is characterized by elements of variation, selection, and dynamism.

## 1. Introduction

The personal practical knowledge (PPK) of teachers, both pre-service and in-service, significantly influences their teaching practice (Connelly et al., 1997; Johnson and Golombek, 2002; Schaefer and Clandinin, 2018; Clandinin, 2019; Wiens et al., 2022). Certain forms of PPK can foster positive professional development by informing pedagogical theories, while others may introduce bias against alternative perspectives (Nunan, 1992; Tsang, 2004). PPK embodies the knowledge and beliefs authentically implemented in education and teaching (Meijer et al., 2001; Chen, 2011, p. 59), representing “a moral, affective, and aesthetic way of knowing life’s educational situations” (Clandinin et al., 2016). As pre-service teachers primarily engage with theoretical pedagogical content knowledge (PCK), it becomes essential to investigate the development and transformation of PPK within such contexts (Wieser, 2016). Specifically, the process of converting theoretical PCK into sustainable PPK warrants exploration.

The impetus for this study is a “blended learning” reform initiative led by an English as a Foreign Language (EFL) teacher-educator researcher. The creation of a blended learning community aimed to provide a broader and more profound platform for student teachers to delve into English pedagogical knowledge, articulate their understandings, and connect their theoretical knowledge with personal experience. This blended learning approach integrated “educator’s lecturing + student teachers’ group presentation+ team microteaching + cloud interaction”.

This research aims to examine how EFL student teachers, within the blended learning community, transmute PCK into their PPK. Theoretical and practical implications are inherent in understanding how PCK is transformed and assimilated into pre-service teachers’ PPK, particularly in relation to their personal experiences.

## 2. Literature review

### 2.1. PPK transformation and development

PCK is characterized as the foundational theoretical comprehension within a teacher’s specific domain, providing the underpinnings of a teacher’s instructional expertise (Shulman, 1987; Meijer et al., 1999). In contrast, PPK is defined as a teacher’s cognizance of the pedagogical strategies they employ, their mastery of subject-specific terminology, and their convictions regarding teaching and learning (Clandinin et al., 2016; Clandinin, 2019; Wei et al., 2023). PPK encompasses a teacher’s knowledge and beliefs about their instructional practices, their understanding of the disciplines they impart, their insights about their students, and their comprehension of their professional and personal contexts. The distinction between PCK and PPK was initially highlighted as a divergence by Dewey in 1904 (Úcar, 2022), signifying the nuanced differences between these two interrelated facets of pedagogical knowledge.

The intersection of PCK and PPK has become a critical area of focus in understanding effective teaching and pre-service teacher education. Both PCK and PPK are instrumental in shaping various aspects of teaching, such as bridging theory and practice, addressing teaching dilemmas, enhancing adaptive expertise, and updating teaching styles (Roblin et al., 2014; Chen et al., 2017; Männikkö and Husu, 2019; Chaharbashloo et al., 2020).

Recent empirical studies have underscored the importance of developing student teachers’ PPK before they embark on their teaching careers (Maaranen et al., 2016; Debreli, 2019; Allas et al., 2020; Chaharbashloo et al., 2020). However, there is a paucity of research exploring the transformation process of PCK into PPK and the acquisition of PPK components.

In their research, Žáčok et al. (2020) contend that both theoretical knowledge and practical skills are necessary. They conclude a reciprocal and interdependent relationship between theoretical and practical knowledge, wherein theoretical knowledge provides a foundation and context for practical knowledge, and practical knowledge enables the application of theoretical knowledge in real-world situations with a focus on variation and innovation. Schiller and Zander (2023) similarly found that while PCK forms a common knowledge base shared by teachers, it is the individual teacher’s PPK, built upon this shared PCK and shaped by personal experience, that creates different interpretations of the teaching content.

Echoing these sentiments, Wolmarans (2022) argues that practical knowledge, in professional education and practice, is not merely an application of theoretical knowledge. It provides specialized, contextually-embedded knowledge needed to solve real-world professional problems. Some of this practical knowledge transforms from theoretical knowledge, while the rest originates from the professional’s past exposure to their profession’s nuances.

In the context of vocational education, Wildeman et al. (2023) found a significant difference in teachers’ PPK. This divergence was attributed to two factors: teachers’ limited reliance on their PCK in teaching and the significant influence of their personal beliefs about teaching and learning on the transformation from PCK to PPK.

Wei et al. (2023) found that the development of practical knowledge in teachers requires facilitation through “formative interventions”, which involve deliberate and systematic actions that disrupt usual processes in teacher education, allowing teachers to reflect on their practices and build practical knowledge. These interventions enable the integration of PCK and PPK in teacher education. Úcar (2022) adds a broader context by asserting that both theoretical and practical knowledge are inseparable and vital in pedagogy. The development of these knowledge types involves a complex interplay across three levels: research, integration, and relational. The transformation of PPK from PCK, according to his study, is greatly influenced by all participants in the blended learning community.

The literature reviewed above clearly indicate that the relationship between PCK and PPK is multifaceted and influenced by a range of factors, including individual experiences, professional exposure, personal beliefs, and the collective learning community. The transformation from PCK to PPK is not a straightforward process but requires deliberate intervention and is significantly influenced by both personal and collective aspects.

The transformation between PCK and PPK can be interpreted from two perspectives: the integration view and the difference view (Wieser, 2016). The integration view posits a continuum where PCK can evolve into PPK in practice. Conversely, the difference view suggests a fundamental distinction between the two, where PPK can be transformed into PCK when brought into focal awareness. However, the integration view cannot fully explain instances where teacher education programs fail to achieve their goals due to a divergence between PCK and PPK (Wieser, 2016). Similarly, the difference view falls short in explaining the process of transforming theoretical knowledge into PPK.

Research on PPK has predominantly focused on in-service and experienced teachers, with less attention given to student teachers (Wei et al., 2023). Existing studies primarily aim at diagnosing rather than understanding PPK cognition and transformation.

Several studies have attempted to identify specific strategies and activities to facilitate the transformation from theoretical knowledge to PPK in teacher education. Allas et al. (2020) suggested that video-based reflection can aid student teachers in gaining new insights into PPK, but the relationship between these new insights and their acquired PCK remains unexplored. Similarly, Debreli (2019) found shadowing techniques effective for PPK enhancement, yet the process underlying this enhancement remains unclear. Concept mapping was useful in raising student teachers’ awareness of acquired PCK and developing PPK (Wei and Lu, 2022), but the mechanisms behind this process and the restraining contextual elements in the learning community are still unclear. As Cain (2015, p.497) aptly noted, “the empirical literature around knowledge transformation is thin and recent empirical studies continually struggle to illustrate how knowledge transformation takes place”.

Úcar (2022) asserts that both types of knowledge are crucial in pedagogy and their development involves a complex interplay at the research, integration, and relational levels. The research level involves the production of theoretical knowledge, the integration level involves the amalgamation of theoretical and practical knowledge, and the relational level involves the knowledge that emerges from the educator-participant relationship. In this context, PCK aligns with the integration level and PPK the relational level, suggesting that the transformation of PCK to PPK is a complex process shaped by all participants in the blended learning community.

### 2.2. PPK development in learning communities

Knowledge communities play a crucial role in refining teachers’ PPK, thereby fostering professional and identity development. The expansive learning theory underscores the significance of PPK development through collaborative practice within learning communities and proposes an open-ended cycle of PPK learning (Engeström, 2015; Engeström and Sannino, 2021; Wei et al., 2023). This cycle, encompassing seven stages, provides a framework for understanding how PPK is cognized and how student teachers’ PCK is examined and transformed into PPK within the context of blended learning communities.

Several studies demonstrate the transformative potential of learning communities. Li et al. (2019) reported that an online teacher community in China facilitated the integration of personal and professional identities. Similarly, Morton and Gray (2010) found that a shared lesson planning conference fostered the effective development of pre-service English teachers’ PPK. Craig (2013, 2014) and Li and Craig (2019) documented the construction and reconstruction of veteran Chinese teachers’ and beginning American teachers’ PPK and identities within online learning communities. These findings suggest that teachers’ PPK can emerge and be reconstructed within learning communities.

As participants in the learning community evolve from sharing theoretical knowledge to exchanging personal narratives (Sarasa, 2015), a shared understanding bridges theory and practice, facilitating the development of PPK. PPK development models, such as those proposed by Nasri et al. (2023) and Mansfield (2023), highlight the effectiveness of partnership, reflective pedagogy, and teacher learning in bridging PCK and PPK within blended learning communities. They concur on the necessity of facilitative interventions in PPK transformation within learning communities, termed as “formative intervention” (Wei et al., 2023).

Despite the theoretical and empirical research on knowledge transformation and PPK development within knowledge communities, there is a need for further focused inquiries into the transformation of PCK into PPK within blended learning communities, particularly in the context of the contemporary information age. This is the focus of the present inquiry.

## 3. Research methods

### 3.1. Research context and questions

This investigation is grounded in the EFL pedagogy course for third-year student teachers in a normal university, where a blended learning community was created to address time and space limitations inherent in the course. The community comprised four modules: an introduction to dominant ELT approaches and L2 acquisition theories by the teacher educator, group presentations where student teachers expounded on their PCK on specified ELT topics, team microteaching sessions where teams practiced teaching different lesson types using high school textbooks, and cloud sharing and discussions. The first three modules were conducted offline, while the fourth module ran concurrently with the other modules throughout the semester. Offline lessons ended with discussions, which then continued online. Students were mandated to share their reflective journals, respond to the teacher educator’s class observation notes, and engage with their peers’ journals online. Their performance in group presentations, team microteachings, and online learning engagement was used for evaluation purposes.

The overarching goal of this study is to discern the mechanism by which EFL student teachers transform their acquired PCK into PPK. The study focuses on the following research questions:

1. What PCK have the student teachers acquired?
2. How do individual student teachers transform this PCK into their PPK?
3. How does the blended learning community facilitate the transformation of PPK?

### 3.2. Participants

The participants were three student teachers from the course with diverse learning experiences and academic backgrounds, none of whom had teaching experience prior to joining the blended learning community. The participants were Ting, who graduated from a distinguished provincial middle school in Southwest China, Qian from a regular middle school in Central China, and Yue from a distinguished provincial middle school in Central China.

Ting and Qian had similar learning experiences in their middle school English classes, characterized by a focus on vocabulary and grammar, traditional lectures, and a teaching model adhering to the “presentation – drills – written exercise” sequence. Their classes were dominated by teacher-led deductive presentations with minimal variety in activities. The students followed a passive “receiving” role, and the classes’ effectiveness was contingent on the students’ motivation to learn and their ability to follow the teachers’ presentations. A minor difference between Ting and Qian’s experiences was that Qian’s received instruction focused solely on vocabulary and grammar, while Ting’s included listening training in a test-like format.

Contrarily, Yue’s experience was markedly different, featuring a teaching model of “picking up the model language – recite – written and oral practice,” diversified teaching and learning activities of both oral and written practice and games, and a learning mode of “note-taking – oral and written practice,” in both individual and collaborative forms.

### 3.3. Data collection and analysis

The research adopted a field-based qualitative methodology, situated within the context of a blended learning community specifically designed for a course in EFL pedagogy, under the instruction of the first author of this paper. A multi-source, qualitative data collection approach—both offline and online—was utilized throughout the course’s progression. This data was then meticulously analyzed on a thematic basis, with a particular emphasis on the transformation of PCK into PPK.

The use of field-based data collection was deemed appropriate for the objectives of our study for a couple of reasons. Firstly, the development of PPK is understood to occur within field practice, as suggested by Mansfield (2023) and Wei et al. (2023). Secondly, field-based data offers an authentic representation of teachers’ PPK development within their regular teaching practice, thus providing intrinsic value to our analysis. We employed a thematic data analysis methodology to deeply explore how PCK is gradually transformed into PPK, and to elucidate the role of the blended learning community in facilitating this transformation.

Data were collected throughout the semester within the blended learning community, covering both offline and online activities. The data types included class observation notes on the student teacher participants’ performance in presentations and microteachings, interviews with the participants, participants’ lesson plans, and their reflective journals and feedback on peers’ journals shared on the cloud platform. The data collected were qualitative. The data were gathered through a concurrent and triangulated methodology. This approach ensures a comprehensive and robust data collection process. Each type of data, providing a holistic and complementary perspective, is systematically utilized to address the three intricately linked research questions of this study.

The data were subject to thematic analysis, oriented by the three-dimensional commonplaces: temporality, sociality, and place, as outlined by Clandinin (2019, 2023) and Clandinin et al. (2016). Temporality, denoting “temporal transitions,” directs our focus towards the evolution of learners’ PPK by establishing connections between past, present, and future experiences. Sociality, representing “social milieus,” enables us to observe the development of learners’ PPK by associating personal conditions with the broader social context. Place, referring to “geographic locations,” permits us to scrutinize learners’ PPK development within specific contexts, such as the blended learning community examined in this study.

In this study, or temporality, data were analyzed to understand how participants’ English pedagogical PPK and understandings evolved over the semester. Participants’ pedagogical beliefs and PPK prior to the course, as well as their plans for future teaching, were also considered.

Under sociality, personal factors such as “feelings, hopes, desires, aesthetic reactions, and moral dispositions” (Clandinin, 2023) were considered to understand how personal narratives influenced participants’ PPK and understandings about effective and useful EFL pedagogy. The impact of middle school teachers, peers, and the current course instructor on participants’ prior PPK, and how these interactions influenced their academic life, was also analyzed. Attention was given to how feedback from external environments interacted with participants’ idealistic beliefs about English pedagogy, that is, the “world travel” between personal and social worlds (Clandinin, 2023).

In terms of place, data were analyzed to examine the mechanism of PPK transformation within the specific context of the blended learning community as “the specific concrete boundary of inquiry” (Clandinin et al., 2016) Meanwhile, data were also analyzed to examine how the blended community facilitated the participants’ PPK transformation.

## 4. Results

The pedagogical journeys of the three student teachers intersected in a single classroom, under the guidance of the same teacher educator. As they project their paths into the future of teaching, a sense of uncertainty and potential for growth is evident (Clandinin, 2023). However, reflecting on their past (Clandinin, 2023), it becomes apparent that their routes to this point have been diverse. They have encountered a variety of teachers and have differing experiences with prior English learning. These differences have significantly shaped their acquisition of PCK and their transformation of PPK.

### 4.1. Acquisition of PCK within the blended learning community

All three participants expressed that they had developed PCK, both in terms of underlying conceptual beliefs and in the design of teaching procedures and activities for various lesson types. As evidenced in their reflective dialogues, they attributed their progress to the collaborative learning activities offered in the course, particularly microteaching (Ting and Yue), peer journal review (Qian), and group presentations (Qian).

Ting noted, “In this course, I absorbed innovative teaching methodologies, strategies and the theories that underpin them, and was granted the opportunity to apply these during micro-teaching sessions” (Ting, reflective journal). Qian reflected on the variances in understanding and approaches to teaching, gained from offline microteaching and online journal reviews, stating, “I acquired theoretical knowledge from you and classmates’ group presentations…, offering me a broader perspective on effective teaching methods and activities. At the meantime, in both offline and online collaborative sharing and learning helped me realize that it was common and normal that we student teachers had different understandings and perspectives about the best ways and activities of teaching.” (Qian, reflective journal). Yue appreciated the practical application and interaction provided by online discussions and offline microteachings, noting, “These opportunities allowed us to deepen our understanding and apply what we learned” (Yue, reflective journal).

While the three participants collectively enhanced their PCK, their individual learning engagements, expectations, and focuses varied, leading to unique learning outcomes within the blended community.

For Ting, the course was an exploration of teaching theories and the rationale behind teaching methodologies. She expected to understand the teaching styles of her previous instructors and their underlying motivations, stating in the interview “to learn the way my former school teachers employed and understand why they taught in that way” (Ting, interview). In the blended learning community, she gained insights into novel teaching designs and their theoretical foundations, surpassing the pedagogy employed by her middle school English teachers. She was particularly struck by the significance of proper classroom teaching objectives and scaffolding activities, which clarified the relationship between teaching objectives and activities. As reflected on her own teaching design, she wrote “I was suddenly enlightened when Professor X analyzed the objectives in my teaching plan” (Ting, reflective journal). Moreover, she realized the necessity of pre-listening and pre-reading activities, a shift from her previous middle-school English learning experience. She narrated in the interview, “In my memory and original understanding, that kind of activities did not exist as my school teachers never employed them, and they just provided us with learning materials and asked us to answer questions directly. In this course, I learned that it was necessary to prepare scaffolds for students before they were required to do a task” (Ting, Interview).

Qian, on the other hand, confronted her entrenched belief in traditional “duck-feeding” methods through differing perspectives on specific teaching issues. Her goal was to acquire both pedagogical knowledge and teaching skills. The course provided a platform for her to meet these expectations by engaging with her peers and receiving feedback from the teacher, thereby promoting her understanding of English teaching. She wrote “I learned a lot from the professor’s feedback as it is focused on what we need to change and improve. We spend much time to figure out the answers to professor’s questions by discussions and searching for academic papers” (Qian, reflective journal). While learning alongside her classmates and teammates, she kept individual and independent thinking. As reflected by her, “we had a lot of face to face and online discussions, even argues. I also read classmates’ online journals. In those discussions and journals, I learned different perspectives in understanding teaching…. But I have been uncertain about the effectiveness of those new teaching ideals and methods, as I have never experience them in my school English learning” (Qian, interview).

Yue found value in the wide range of teaching activities available in the community and developed a deeper understanding of teaching reading. Her educational background had already exposed her to innovative teaching designs, thus this course served as a continuation of her teaching learning journey. Her narrative about reading instruction illustrates her evolving comprehension of lesson planning and the flexibility required in teaching. “I had no idea about how to write a reading lesson plan when we were supposed to do that in the Public Pedagogy course I took last semester. Then I found the general “pre-while-post reading” framework from the internet. Later on, when we did the group presentation in this course, I began to acknowledge that different stage of a reading lesson should be realized by different activities.… When we did the microteaching, I realized that the activities should not be fixed, and they should be chosen and arranged flexibly to serve the objectives of different teaching steps respectively” (Yue, interview).

The disparity in learning outcomes suggests that while Ting and Qian primarily focused on gaining PCK, their learning was concentrated on pedagogical knowledge acquisition and understanding. In contrast, Yue placed greater emphasis on applying knowledge in lesson planning and microteaching, thereby transitioning part of her theoretical PCK into her PPK. A comparative analysis of the three participants reveals a connection between their past educational experiences, course expectations, and learning outcomes, which will be further explored in the discussion section.

### 4.2. Transformation from PCK to PPK

PCK can be either theoretical or practical (Úcar, 2022). However, it does not evolve into the student teachers’ PPK until it has undergone transformation. In this transformation process, some aspects of PCK are embraced, while others are disregarded or neglected. The mechanisms through which acceptance or rejection occurs are distinct for each individual, as demonstrated by the three pre-service teacher participants in our study.

Ting’s learning primarily focused on the design, arrangement, and functionality of English teaching activities. She first familiarized herself with the activities relevant to different types of lessons. Subsequently, she evaluated their strengths and weaknesses, drawing on both the accompanying lecture materials and her personal experiences. Ultimately, she concluded that the purpose of teaching activities is to facilitate the achievement of teaching objectives. As she narrated, “I got familiar with some teaching activities first in the course, for example, …. Later, I came to understand the functions of various teaching activities, along with their strengths and weaknesses in the presentations and discussions. My current understanding is that the design and arrangement of activities should be oriented towards achieving the teaching objectives of different steps” (Ting, interview).

Ting recognized and accepted the teaching methodology characterized by student-centered activities and tasks, as advocated by the teacher educator. She expressed her intention to incorporate these methods and activities into her future teaching in the interview, “I am going to use them in my future teaching.” However, she also expressed concerns about acceptance and understanding from her future professional community, as the student-centered teaching approach contradicted her familiar school learning experiences. She narrated in the interview, “I think it is important to reach the school and parents and obtain their acceptance if we want to make some change in teaching” (Ting, interview, reflective journal).

Qian’s experience of accepting PCK and transforming it into her PPK was unique. Most of the PCK she acquired was entirely new and conflicted with her previous school learning experiences, leading her to “feel uncertain and even resistant” (Class observation notes). Her transformation process was marked by tension, conflict, doubts, and instability. Initially, she rejected the course’s advocacy for students to learn English through various activities and tasks, as it contrasted starkly with her experience of teacher-led learning. Qian initially viewed these activities as mere “trimmings.” The tension and resistance in Qian’s new PCK acquisition and PPK transformation were reflected in the interview, “My school English teachers used traditional teacher presentation-dominated teaching method. They scarcely employed activities in class. I think activities are just external packings in teaching. Why should we bother with those packings?… The direct presentation and explanation were clear and efficient. Some students, like me, prefer teacher’s direct teaching of language points and did not want to do those activities. But for other students, the sole presentation and explanation mode might be too boring….” (Qian, interview).

However, over time, Qian’s understanding evolved. She came to appreciate the integration of various teaching activities, as she gradually understood that the integration of various teaching activities and teaching content were different from the traditional teaching not only in their superficial appearance, but also in their function and effectiveness, as narrated in the interview, “though I thought the activities are just outer packings, the employment of them in teaching does have effects. The sole teacher’s presentation is limited in its effectiveness. In microteaching, some of my classmates designed and used various activities very well, the teaching effects were good. So now I do not think those activities are just non-compulsory time-consuming packings. Idealistically, students should love them” (Qian, interview). But the hesitation and reluctance in the interview showed that Qian’ acceptance of the newly acquired PCK was not without reservation, and the new teaching methods and ideals had not been transformed into her PPK as she was still doubtful about their feasibility. Bearing doubts, she did not completely reject them either. The tension and uncertainty between understanding and acceptance were obvious, due to the conflict between her former familiar school learning experience and the advocacy in the course as well as the positive effect of her peer’s practice in microteaching. She stated, “Maybe I will try them out when I become a teacher in future to see if they really work to my students, but right now I’m still not sure as personally I did not have that experience after all in my middle school English learning (Qian, interview).

Yue’s learning experience in the course emphasized that language skills and strategies should be both explicitly and implicitly taught and trained in various types of lessons. This contrasted with her prior school English learning experience, which emphasized memorizing and accumulating model language expressions. Despite this discrepancy, Yue was receptive to the new approach and acknowledged the importance of strategy teaching. She intends to include language skills training and strategies in her future teaching, as she narrated, “I think we should put what we learned in this course in use in middle school English teaching, for example, to teach how to read. Those strategies and skills need to be taught in class because students need them. I’m going to employ them in my future teaching” (Yue, interview).

In Yue’s case, the new theoretical PCK she acquired in the blended community has been transformed into her PPK. This successful transformation may be partially attributable to her deep and comprehensive understanding of the new theoretical knowledge, and partially to her recognition of her deficiencies in certain language skills and the necessity of their inclusion in school English teaching. As indicated in the interview, “I spent a lot of time on learning reading teaching in this course. The more I know, the more I realize the importance of reading skills and strategies in teaching. I was not good at listening and could not get a fair understanding in listening class at the beginning stage of my college. I felt anxious but no one helped me with that, and my middle school teachers did not train it either.” (Yue, interview).

### 4.3. The role of the blended community in facilitating transformation

The narratives and reflections of Ting, Qian, and Yue reveal that their transformation of theoretical (PCK) into PPK is influenced not only by their prior experiences, but also by the milieu wherein they interact with others. This milieu has been referred to as the “professional knowledge landscape” (Clandinin, 2023), “interactive context,” or “community of truth” (Palmer, 1998). In the context of this study, the transformative environment is the blended learning community established for the ELT Pedagogy course.

The blended community functions as a conduit for transformation, fostering an expansive and profound platform for collaborative learning and mentoring. This platform evolves as a multifaceted dynamic system, spawning opportunities for learners to transform theoretical knowledge into personalized PPK. The constituents of this complex system include student teachers, the teacher educator, the physical classroom, the cloud-based platform, the theoretical PCK imparted during lectures, educator’s observational notes and comments, student teachers’ shared ideas, lesson plans, reflective journals, both offline and online comments, co-built resources, and the interactional dynamics among these elements.

The blended community catalyzes knowledge transformation in two principal ways. Firstly, peer sharing and discussions deepen the understanding of PCK among student teachers. Most online journals focus on specific topics. “Coupled with peer responses, they offer a rich resource for learners to explore unfamiliar territories” (Class observation notes). As Ting reflected, “I gleaned substantial knowledge from my classmates’ cloud-based journals. Some of their ideas remain novel to me even as we near the end of the course” (Ting, reflective journal).

Secondly, the blended community extends the boundaries of PCK beyond the confines of the teacher educator’s delivery, thereby “broadening the scope and viewpoint” (Class observation notes). The diversity of ideas and resources, both within and outside the classroom, enable student teachers to decode PCK from multiple angles, referencing varied application contexts. As reflected in their conversations, “By reviewing your class observation notes and comments shared on the cloud, I discovered aspects and details of teaching that I had overlooked but you had noted… I believe my reflections and thoughts, triggered by my classmates’ journals or comments, are beneficial as they prompt me to think more and understand more deeply” (Qian, interview). “One of Y’s journals focused on ‘continuation writing’. I was unfamiliar with it and intrigued to learn more. My exploration led me to understand the necessity of bridging reading with writing in teaching… My classmates’ online discussions prompted me to reassess my understanding and adopt a different perspective” (Yue, interview).

The blended learning community nurtures critical thinking among student teachers regarding their previous learning experiences and facilitates the contextualization of PCK. It offers opportunities for the collation, cross-checking, reflection, and transformation of varied understandings of PCK based on individual experiential backgrounds. The breadth and depth of theoretical PCK are interpreted and accepted differently by each student teacher, as expressed in their journals and feedback. The tensions between the student teachers’ prior English learning experiences and their peers’ and their own understandings stimulate reflection and further inquiry, driving forward their ongoing knowledge transformation and PPK development. The potency of this transformative mechanism is reflected in the participants’ reflections. “I find professor’s offline and online comments invaluable in understanding and evaluating our former school teachers’ teaching and classmates’ microteaching…. The journals and feedback are equally beneficial as they provoke us to reflect on our own interpretations and learn from our classmates” (Ting, reflective journal). “During the microteaching session, I was oblivious to some of the innovative designs of other teams until I read professor’s comments and those of my classmates. This prompted me to recall the details, understand their nuances and the rationale behind them, and contemplate on how I could incorporate them into my teaching” (Yue, reflective journal).

## 5. Discussion

### 5.1. Selective transformative cognition of PPK

As evidence from the preceding section suggests, the participants have demonstrated a partial and selective assimilation of the principles and practices introduced in the course. Such selective transformation from theoretical PCK to their PPK is significantly influenced by their learning expectations and prior experiences.

When observed collectively, the transformation from PCK to PPK amongst the three participants is uniformly selective and partial. This selective transformation is governed by the interplay of their previous learning experiences, course expectations, and engagement in learning. The dynamics of these factors determine the types of PCK likely to be transformed into PPK.

Examined individually, each student teacher’s transformed PPK concentrates on different facets of PCK. Their learning expectations differ, and thus the magnitude and breadth of transformation also vary. This transformation is conditioned by distinct modes of interaction within the learning community.

Ting’s expectations, which are vaguely oriented towards understanding her previous English learning experience, can be classified as ‘backward-looking’ expectations. These expectations did not provide a potent motivation for engagement in learning innovative teaching methods in the course. This is reflected in her relatively passive classroom performance and involvement in the blended learning community. Ting’s transformed PPK primarily encompasses understanding and evaluating the teaching methods employed by her school English teachers. The transformation is selectively partial, given her limited ‘backward-looking’ expectations and lack of strong learning motivations.

Qian’s PPK transformation primarily focuses on teaching skills and her own experience in group presentations and team micro-teaching. She hoped to acquire specific teaching skills and observe how the educator presents PCK. Her “present-sitting” expectations, focusing on PPK development and personal experience in experimenting with teaching skills, provided her with moderate motivation to learn new pedagogical knowledge. Qian’s PPK transformation was tentative, primarily about teaching skills, especially presentation skills. The transformation was selectively partial despite her clear expectations, due to her skepticism about its effectiveness.

Yue expected the course to pave the way to a future teaching career, aiming at acquiring practical teaching skills. With very specific expectations, she actively engaged in the blended learning community by answering questions, initiating discussions, sharing in-depth thematic journals, and providing interactive feedback to peers’ comments. Unlike Qian, Yue’s middle school English teacher employed diverse teaching activities, which fostered her openness to innovative activities. Yue’s transformed PPK primarily included practical teaching skills and activities. Despite her clear ‘forward-looking’ expectations and uninhibited by prior learning experiences, Yue’s transformation of PCK into PPK was still selectively partial, as it was confined by the scope of her expectations.

Swart et al. (2018) posited that language teachers’ PPK “originates from their professional practice and is based on their past experience, current awareness, and future expectation.” The findings of the present inquiry substantiate their argument. The past experience herein includes the prior school English learning experience, current awareness can be interpreted as the PCK acquired in the blended community, while future expectation can be interpreted as course expectations.

As discussed above, clear and specific expectations stimulate learners’ engagement, and the scope of these expectations directs its focus. However, the nature of learning expectations and the degree of learning engagement do not necessarily correspond to the extent and depth of PPK transformation. This is because it is conditioned and filtered by the learners’ prior experiences, as exemplified in Qian’s case.

This selective process results in the diversity and difference in student teachers’ PPK development, echoing findings from previous research. Both qualitative (Meijer et al., 1999) and quantitative studies (Meijer et al., 2001) found that while there was shared knowledge among teachers, a wide diversity and significant difference existed in their PPK regarding teaching reading comprehension.

Aside from course expectations, the selective transformation of PPK from PCK among student teachers can also be attributed to agent differences in knowledge production (Úcar, 2022). While PCK can be a result of individual or collective integration, PPK arises primarily from singular relationships. The PCK acquired by student teachers is collectively provided by the teacher educator in the course.

When PCK is transformed into PPK, a process of “functionally asymmetric negotiation” occurs (Úcar, 2022). This negotiation takes place among various entities: between the teacher educator and individual student teachers, among the student teachers themselves, and between an individual’s past experience and the newly acquired PCK within the blended learning community. This asymmetric negotiation in the course learning experience may lead to the selective transformation of PPK, as evidenced in previous research (Noroozi et al., 2018; Noroozi, 2022). Consequently, the transformation of PCK into PPK is not a uniform process, but one that varies based on individual experiences and interactions.

### 5.2. Mechanism of filtering in PPK transformation

The transformation of PCK into PPK is a process that all three participants underwent, albeit with varying degrees of transformation. Crucially, not all theoretical PCK acquired was transformed into their PPK. This transformation process is shaped and filtered by their learning expectations, learning performance, prior experiences, and the beliefs derived from these experiences.

This filtering mechanism is particularly evident in Qian’s case. Qian recognized that the PCK advocated in the course differed from the teaching methods of her middle school English teachers. While she acknowledged their alignment with the New Curriculum prescriptions (MOE, 2018) and their relevance for contemporary English teaching pedagogy, she retained skepticism about their effectiveness. Her previous English learning experience, characterized as a “direct duck-feeding” mode, led her to believe that teaching effectiveness is more a result of learners’ voluntary engagement rather than teaching methodology. Her personal success in gaining admission to a prestigious university reinforced her belief in the efficacy of traditional teaching methods and fueled her doubts about the feasibility of the innovative teaching methods introduced in the course. Consequently, she attempted to understand them but was reluctant to accept and potentially utilize them in her teaching.

In the journey of transforming PCK into PPK, doubts and uncertainties inevitably emerge due to the gap between prior experiences and newly acquired knowledge. These doubts initiate tensions that create opportunities for knowledge transformation. Whether this transformation occurs or not is contingent on the individual’s willingness and strategies to resolve these tensions.

In contrast to Qian, Yue demonstrated a more open attitude towards the innovative PCK introduced in the course, hence her approach to dealing with the ensuing tensions differed. Both Qian and Yue initially expressed uncertainty about the effectiveness of activity-based teaching introduced in the course, leading to a tension between their prior experiences and the new PCK. However, their strategies for easing this tension diverged. Qian did not actively engage in in-depth discussions to dissolve the tension, whereas Yue attempted to find solutions by actively participating in multiple rounds of discussion and commenting in the blended learning community.

The disparity in learning participation between Qian and Yue correlates with their respective transformations of PPK. PPK, as a teacher’s construct, is narratively embodied and practically expressed (Schaefer and Clandinin, 2019a; Talaee et al., 2023). The narrative and practice in this study are derived from the participants’ learning experiences within the blended learning community. While there are shared elements in their experiences, such as the PCK provided by the course instructor and class assignments, the learning experiences of each participant are distinct, largely due to their engagement in different collaborative groups. Consequently, their participation within the learning community varies, leading to differences in the content and magnitude of PPK transformation.

The variation in PPK transformation among individual student teachers, such as Qian and Yue, aligns with findings from Golombek (2009) and Xu and Connelly (2009). These studies concluded that teachers’ cognition of PPK is accompanied by, and filtered through, their participation in professional development activities. Additionally, the unique participation of each student teacher may involve varying degrees of “interpersonal sensitivity” (Spoel et al., 2020), which can subsequently influence the transformation of their PPK.

The distinct approaches of Qian and Yue in addressing these tensions may be linked to their differing prior learning experiences. Qian, who had succeeded in gaining admission to a key university despite her teachers’ exam-focused, “duck-feeding” teaching approach, held a strong belief in this method and was resistant to alternative effective teaching methods. Conversely, Yue’s exposure to various learning experiences and diverse classroom activities during her schooling, as well as her experience with “group cooperative inquiry learning,” may have developed her ability and mental readiness to answer questions through inquiry. These experiences likely facilitated her open attitude towards the new PCK and her ability to dissolve tensions by embracing them, resulting in a less filtered transformation from PCK to PPK.

The significant influence of prior learning experiences on a teacher’s PPK development has been substantiated in various studies. Li (2020) established that PPK is shaped by teachers’ experiences within both formal and informal educational spheres, including their personal life experiences. Similarly, Wang et al. (2018) discovered that both pre-service learning and in-service teaching experiences can significantly impact PPK development. Furthermore, Schaik et al. (2018) found that one’s academic background can either facilitate or impede the cognition of PPK. These findings collectively underscore the pivotal role of prior learning and experiential contexts in shaping the development and understanding of a teacher’s PPK.

Examining across all participants, the filtering mechanism in knowledge transformation is influenced by both internal components within the learning community and external considerations. The internal components include the participants’ and their peers’ prior English learning experiences, varying interpretations of these experiences, diverse beliefs about English learning, and differing learning expectations. These internal components create a unique filtering mechanism for each individual, affecting the extent and focus of knowledge transformation. The external factors include the relevance of theoretical PCK to future teaching practice and the evaluation methodologies of future teaching. The former refers to the extent to which theoretical knowledge is directly related to teaching practice. That explains why limited volume of theoretical knowledge on second language acquisition, which is a compulsory session, was transformed into the participants’ PPK, as indicated in their reflective journals, comments and interviews. The latter refers to how routine teaching is evaluated in examinations, such as in college entrance exams. That explains why PCK closely related to the content tested in exams was attended to and transformed more into the participants’ PPK.

The mechanism that governs the acceptance and transformation of PCK into PPK functions in complex ways. In some scenarios, internal components exert a more significant influence, whereas in others, external factors prevail. The development of a teacher’s PPK is the outcome of the interplay between these internal and external components. This is consistent with Huang’s (2010) research, which found that EFL teachers’ PPK about grammar teaching is shaped by the interaction of multiple sources from their previous experiences as EFL learners and teachers, along with the unique socio-educational context of the EFL setting.

The relationship between prior experience and selective knowledge transformation identified in this study aligns with prior research. Baleghizadeh and Shahri (2014) found that teachers’ divergent understandings about English speaking competence and teaching reflect the “interwoven nature of learning experience with teaching conceptions.” Schaefer and Clandinin (2019b) discovered that novice teachers’ “stories to live by were shaped in their early personal knowledge landscapes and embodied in their PPK.” In their study, Ferry et al. (2022) discovered that PPK cognition is significantly shaped by the experiences of student teachers prior to and during their teacher education. Moreover, they highlighted that the acquisition of PCK during teacher education serves as an even more potent influence, exerting a substantial degree of shaping power. However, certain external components can exert a stronger influence on shaping teachers’ PPK in a particular socio-educational context, as found in Huang’s (2010) study, which demonstrated that teachers exhibited similar classroom grammar teaching practices despite their diverse backgrounds.

The relationship between external components and PPK transformation identified in this study is congruent with previous research. Huang’s findings suggest that the socio-educational context in Taiwan can significantly shape EFL teachers’ PPK in a similar way (Huang, 2010). Moreover, teaching assessment methods can also influence and shape teachers’ selection and PPK development. In Tang’s (2010) exploration of teachers’ knowledge construction, they found that teachers effectively integrated Assessment for Learning into PCK, transforming it into their PPK as “practicalizing theoretical knowledge,” in conjunction with their complex personal prior experiences. This implies that the transformation of PCK into PPK occurs within a particular social context. The predominant form of teaching assessment in that context can shape student teachers’ judgement about which PCK knowledge could be useful and feasible, thereby influencing their acceptance and transformation of this knowledge, while rejecting others. This might account for Qian’s reluctance and doubt in accepting the innovative PCK and transforming it into her PPK.

### 5.3. Dynamic process of PPK development

The findings of this study unveil a dynamic process wherein preservice teachers transform PCK into their PPK. This process involves not a static but an evolving development of PPK. The transformation begins with familiarization with PCK, progresses towards gradual understanding and acceptance, and culminates in the adjustment and revision of the knowledge in practice. This process situates PCK in specific contexts, thereby imbuing its generic features with the characteristics of particular situations. This specification process fuels the dynamic transformation.

The dynamic process of PPK transformation and development manifests in the continuity and interaction of student teachers’ experiences within the blended learning community. As Clandinin (2023) posits, continuity and interaction are the two criteria that shape individuals’ experiences, which manifest as narratives enacted in specific contexts. PPK emerges from teachers’ “stories to live by” (Connelly and Clandinin, 1999) and evolves in the cyclical process of “living,” “telling,” “retelling,” and “reliving” (Clandinin et al., 2016). In this continuous cycle, teachers’ understandings of their narratives are persistently evolving. Hence, the transformation from PCK to PPK and the ongoing development of PPK are in a constant state of dynamic change.

This dynamic process is corroborated by both previous empirical research and the present investigation. In a study exploring Taiwanese EFL teachers’ beliefs about grammar teaching and PPK, Huang (2010) identified an “ongoing process of change” in cognition and PPK among participants. Similarly, a study by Desrochers (2017) on Canadian preservice teachers’ PPK on student diversity found that the teachers came to understand how their PPK had been shaped and could be reconsidered and reshaped in light of new experiences. In the current study, Yue’s experience of PPK transformation and further development was accompanied by a dynamic process of knowing, understanding, and flexibly applying knowledge. Prior to this course, Yue was unsure about effective reading teaching methods. During the course, she was introduced to various activities for teaching reading, though initially she did not understand the need to use different activities at different stages. Through interaction in the blended learning community, she came to understand that activities should be chosen and designed flexibly to meet the objectives of different stages. Ultimately, she successfully implemented these activities in team microteaching.

The dynamic nature of PPK transformation and development is closely linked to the learning or working community of the teachers. In the current study, the participants’ PPK transformation was guided by the PCK ideals presented and advocated by the teacher educator in the blended learning community. This finding echoes previous research exploring the relationship between teachers’ PPK development and its context. For example, studies by Craig and her colleagues found that the PPK of rural Chinese school teachers varied and developed as the test-oriented and professionally challenging environment changed (Li and Craig, 2019; Zhong and Craig, 2020). A strong connection was found between American school teachers’ shifting identities and changing school landscapes, with teachers’ “stories to live by” potentially becoming their “stories to leave by” (Craig, 2013, 2014).

The dynamism inherent in the transformation process is evident in both the content of PPK and its sources, which are intricately intertwined in an interactive manner. This study revealed distinct patterns of PPK transformation among different teachers. For instance, Ting’s transformation predominantly pertains to classroom teaching activities, with an emphasis on aspects such as design, arrangement, and functionality. Qian’s PPK transformation, however, is centered around the evolving cognition of innovative teaching ideals and the effectiveness of classroom teaching activities. In contrast, Yue’s PPK transformation is more comprehensive, spanning teaching activities, content such as learning strategies, and pedagogical approaches. These variations in the content of transformed PPK are associated with their respective sources. This observation is consistent with the findings of Ferry et al. (2022), who reported that the experiences student teachers gain during their teacher education have the most profound impact on their PPK, particularly regarding instruction and interactions with students in school. Meanwhile, personal experiences before embarking on teacher education significantly influence their PPK about the classroom context and their roles as teachers. Similarly, Chaharbashloo et al. (2020) highlighted the dynamic alignment between different types of PPK and their sources.

## 6. Conclusion

This study embarked on an exploration of how preservice teachers acquire PCK within a blended learning community, the manner in which they transform PCK into their PPK, and the role of the community in shaping this transformation.

It was determined that all participants acquired PCK in relation to conceptual teaching ideals and lesson designs. However, differences were observed both in focus and acceptance of PCK, and in the degree and scope of transformation from PCK to PPK. The transformation and progression of PPK transpire through a selective and dynamic process, characterized by a prevailing filtering mechanism and accompanied by tension. The variance in transformation is influenced by the internal components of the blended learning community, such as prior middle school English learning experiences and course expectations, as well as external conditions, such as teaching evaluations. While the blended community shapes the PPK transformation, it concurrently establishes a dynamic complex system. In this system, the construction of collaborative resources based on peer interaction engenders opportunities for the collision and reflection of diverse understandings, thereby facilitating individual preservice teachers’ PPK transformation and further development.

The findings of this investigation carry implications for teacher educators in designing and delivering methodology courses to augment student teachers’ construction of PCK and PPK. As Rowan et al. (2021) emphasize, it is crucial to teach for diversity. The preservice teachers’ previous school English learning experiences should be considered when designing learning activities and tasks, so these experiences can serve as bridges, enabling learners to connect theoretical knowledge acquired in the community with their prior experiences. An orientation should be incorporated into the course design to elicit clear, specific, and forward-looking learning expectations from the preservice teachers, fostering more fruitful achievements. It is vital to provide appropriate guidance to avoid right-or-wrong binary judgement on theoretical knowledge when preservice teachers interact in the blended learning community, enabling them to discuss from multiple perspectives while maintaining the integrity and depth of the learning community. While offline and online activities and tasks can be integrated within a blended learning community, it may be advantageous to differentiate the focus of these activities to some extent, ensuring that both the immediate and delayed temporal features of the two modes are optimally leveraged.

## Data availability statement

The raw data supporting the conclusions of this article will be made available by the authors, without undue reservation.

## Ethics statement

The studies involving humans were approved by School of Foreign Languages, Central China Normal University. The studies were conducted in accordance with the local legislation and institutional requirements. The participants provided their written informed consent to participate in this study. Written informed consent was obtained from the individual(s) for the publication of any potentially identifiable images or data included in this article.

## Author contributions

QX: Conceptualization, Formal analysis, Funding acquisition, Investigation, Methodology, Project administration, Resources, Software, Supervision, Validation, Visualization, Writing – original draft, Writing – review & editing. TW: Data curation, Formal analysis, Investigation, Methodology, Writing – review & editing.

## Funding

The author(s) declare financial support was received for the research, authorship, and/or publication of this article. This research was funded by National Planning Office of Philosophy and Social Science, grant number 18BYY097.

## Conflict of interest

The authors declare that the research was conducted in the absence of any commercial or financial relationships that could be construed as a potential conflict of interest.

## Publisher’s note

All claims expressed in this article are solely those of the authors and do not necessarily represent those of their affiliated organizations, or those of the publisher, the editors and the reviewers. Any product that may be evaluated in this article, or claim that may be made by its manufacturer, is not guaranteed or endorsed by the publisher.
